# Sex differences in gene regulatory networks during mid-gestational brain development

**DOI:** 10.3389/fnhum.2022.955607

**Published:** 2022-08-17

**Authors:** Victor Hugo Calegari de Toledo, Arthur Sant'Anna Feltrin, André Rocha Barbosa, Ana Carolina Tahira, Helena Brentani

**Affiliations:** ^1^Departamento e Instituto de Psiquiatria, Faculdade de Medicina FMUSP, Universidade de São Paulo, São Paulo, Brazil; ^2^Laboratório de Psicopatologia e Terapêutica Psiquiátrica (LIM23), Faculdade de Medicina FMUSP, Universidade de São Paulo, São Paulo, Brazil; ^3^Centro de Matemática, Computação e Cognição, Universidade Federal do ABC, Santo Andre, Brazil; ^4^Lieber Institute for Brain Development, Baltimore, MD, United States; ^5^Laboratório de Expressão Gênica, Departamento de Parasitologia, Instituto Butantan, São Paulo, Brazil

**Keywords:** neurodevelopmental disorders, sex differences, fetal brain development, gene regulatory networks, systems biology, autism spectrum disorder (ASD)

## Abstract

Neurodevelopmental disorders differ considerably between males and females, and fetal brain development is one of the most critical periods to determine risk for these disorders. Transcriptomic studies comparing male and female fetal brain have demonstrated that the highest difference in gene expression occurs in sex chromosomes, but several autossomal genes also demonstrate a slight difference that has not been yet explored. In order to investigate biological pathways underlying fetal brain sex differences, we applied medicine network principles using integrative methods such as co-expression networks (CEMiTool) and regulatory networks (netZoo). The pattern of gene expression from genes in the same pathway tend to reflect biologically relevant phenomena. In this study, network analysis of fetal brain expression reveals regulatory differences between males and females. Integrating two different bioinformatics tools, our results suggest that biological processes such as cell cycle, cell differentiation, energy metabolism and extracellular matrix organization are consistently sex-biased. MSET analysis demonstrates that these differences are relevant to neurodevelopmental disorders, including autism.

## 1. Introduction

Sexual dimorphism in the context of brain structure, function and chemistry has been consistently reported. Total and regional volume, for example, differs between males and females, including during fetal development (Ritchie et al., 2018; Galjaard et al., 2019). Moreover, males present higher connectivity between different regions, while females demonstrate higher connectivity within regions (Gur and Gur, 2016). Several human disorders affecting the central nervous system are sex-biased in prevalence, age of onset, pathophysiology and etiology, especially neurodevelopmental disorders (Loke et al., 2015). Accordingly, when compared to females, males have a two to four increased risk for neurodevelopmental disorders (NDs), such as intellectual disability (ID), autism spectrum disorder (ASD), attention deficit hyperactivity disorder (ADHD), and early-onset schizophrenia (May et al., 2019). During fetal brain development, sex differences might emerge from the gene regulatory effects of the interplay between gonadal hormones, sex chromosomes and sex specific responsiveness to environmental factors, which collectively might contribute to higher vulnerability in male pregnancies by affecting growth and survival rate (Bale, 2016). Indeed, previous studies have described the association between males' lower adaptability to gestational stress and neuropsychological development deficits (Fink et al., 2018). These differences between males and females are partially attributed to differences in DNA methylation and sex chromosomes transcription factors (TFs), both being associated with the regulation of autossomal genes related to neurodevelopmental disorders (Maschietto et al., 2017; Tahira et al., 2018). In fact, TFs are mediators of cell responses to environmental stimuli and therefore are critical to proper brain development, being associated with multiple NDs including ASD (Santos-Terra et al., 2021).

Although sex differences in neurodevelopment and male susceptibility to NDs have been thoroughly studied, its role in the regulation of multiple collaborating developmental processes remains unclear. The sex bias on gene expression exhibits an overall small effect, suggesting the difference between sexes to be subtle, especially in autosomes (Oliva et al., 2020). Genes encoded in sex chromosomes figure prominently amongst the most differentially expressed between males and females, with the Y chromosome genes displaying the largest differences (Reinius and Jazin, 2009; Kang et al., 2011; Shi et al., 2016; Werling et al., 2016). During the fetal neurodevelopment, sex differences in gene expression occurs mainly in the lateral and medial prefrontal cortex and are associated with pathways altered in some NDs, such as ASD and schizophrenia (Kang et al., 2011; Shi et al., 2016). In addition, it has been described that fetal differentially expressed autosomal genes are more expressed in males and are associated with several NDs, specially ASD, and biological processes such as cell cycle and cell adhesion (Reinius and Jazin, 2009; Shi et al., 2016). Curiously, there are evidences suggesting that genes on interacting pathways of sex-biased genes in fetal brains are more related to NDs risk than the differentially expressed genes themselves (Werling et al., 2016).

The enormous quantity of data obtained from the multi-omics technologies required researchers to analyze the complex interactions between multiple molecules, giving rise to the systems biology field of study (Veenstra, 2021). Complex Network analysis is a systems biology approach to model interactions between biological entities such as co-regulated genes and its regulatory elements (Mähler et al., 2017). Co-expression networks are built based on correlation between the gene expression across samples, and the clusterization of co-expressed genes could indicate that they have related biological functions or participate in the same regulatory pathways (Langfelder and Horvath, 2008). There is evidence of differential patterns of regulation even when genes are not differentially expressed (Gaiteri et al., 2013; Lopes-Ramos et al., 2020). Within this framework, integrative methods that combine co-expression networks with other data types such as protein-protein interactions (PPI) and regulatory mechanisms are not only more informative (Simões et al., 2015), but they also provide more specific results about the imputed biological data (Michoel et al., 2009). These integrative network approaches have been successfully applied to understand gene regulatory differences between males and females in different scenarios (Michoel et al., 2009; Lopes-Ramos et al., 2018, 2020).

We hypothesized that the application of systems biology approaches to the understanding of sex differences in fetal brain development could unravel novel interactions and biological mechanisms underlying sex bias in neurodevelopmental disorders. Our goal was to find differentially regulated biological processes between males and females occurring during the mid-gestational period of brain development, when corticogenesis is the major histo- and neurogenic event. Moreover, we aimed to find signaling pathways and their effectors that were pertinent to these differences. In this context, we applied Complex Network tools and integrated their results to investigate autosomal gene co-expression and regulation in the brain from females and males human fetuses. We removed the genes on the sex chromosomes in order to increase the autosomal gene expression signal (Glass et al., 2014). Then, we conducted a primary analysis using the R package CEMiTool (Russo et al., 2018), which identifies co-expression modules (CM) based on gene expression correlation and reports an first overview of sex differences in module activity. Later, we applied the netZoo package to integrate PPI and motif data, creating regulatory networks for each sex and then comparing their community structure (Glass et al., 2013; Padi and Quackenbush, 2018). Next, we examine how TFs and genes are clustered based on their importance for the modularity of each created module (M), identifying the drivers of regulatory networks sex differences. Finally, we searched for enrichment of these modules for biological functions, cell types, and neurodevelopmental and psychiatric disorders. When combined, these results show differences between sexes in fetal brain development mechanisms and hint at some of its possible effectors.

## 2. Materials and methods

**Figure 1:**
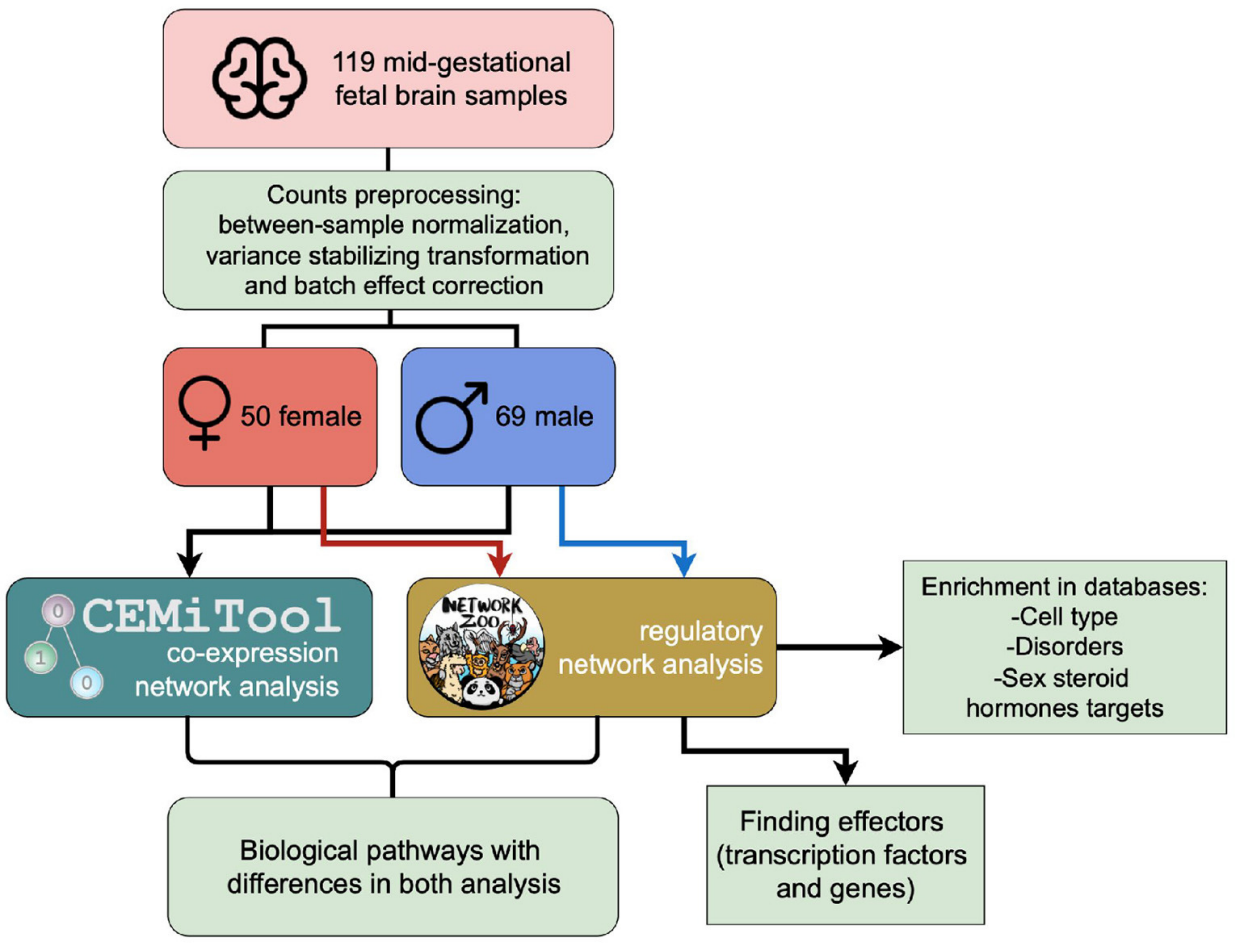
Workflow of the analyses that were carried out using CEMiTool and netZoo with male and female RNAseq data from fetal brain samples.

A workflow chart summarizing the conducted analyses in this study can be seen in Figure 1.

### 2.1. Dataset and pre-processing

RNA-seq data from 120 undissected fetal brain tissues collected from elective abortions were obtained from a public repository (O'Brien and Bray, 2018). The libraries were prepared from total RNA using the TruSEqStranded Total RNA Library Prep kit (Illumina), followed by ribosomal RNA depletion. A minimum of 100 million read pairs per sample were sequenced on Illumina HiSeq systems. Fetal sex was determined both by karyotyping and chromosomal sex genes expression. The dataset consisted of gene expression data from 70 males and 50 females with age ranging from 12 to 19 post-conception weeks (PCW), which were equally distributed for the known variables: age, RNA integrity number and read count (Supplementary Table S1).

The following analyses were conducted using the R language (v4.1.0). Counts for 29,875 genes were provided with between-sample normalization using the median of ratios method and variance-stabilizing transformation (Love et al., 2014), followed by a filter that removed low expressed genes. Additional details regarding the sample processing protocols and the RNA-seq pre-processing are published in the original article (O'Brien et al., 2018). As recommended for co-expression network construction (Langfelder and Horvath, 2008), genes with variance smaller than 0.1 were excluded. Hierarchical clustering based on the Euclidean distance of gene expression abundance demonstrated that one sample was an outlier. We removed this sample and further analyses were performed with the remaining 119 samples. Given its contribution to data variance, batch effect was corrected using LIMMA (v3.48.3) (Ritchie et al., 2015; Supplementary Figure S1). Gene annotation was converted to Gene Symbol^1^ with the package biomaRt (v2.48.3) (Smedley et al., 2009), and 18,904 genes remained.

### 2.2. Co-expression network modeling

The CEMiTool package (v1.16.0) (Russo et al., 2018) was used for the construction of co-expression networks and their clustering into co-expression modules (CMs). CEMiTool is an automated method that creates co-expression networks by intuitively selecting a soft-thresholding power based on the pairwise correlation between genes. This analysis was conducted on the full dataset, without sex stratification. Automatic filtering of genes with low variance using a model of negative binomial distribution to correct for mean-variance dependency and select variable genes and VST were turned off due to previous RNA-seq data pre-processing steps. In order to evaluate the activity of each CM in males and females, samples were annotated according to sex and then submitted by CEMiTool to a gene set enrichment analysis (GSEA) (Subramanian et al., 2005). Genes from each individual CM found by CEMiTool had their expression normalized using z-scores, and these values were used to rank the expression in male and females to perform the GSEA. These z-scores are averaged and corrected by the number of genes to calculate a normalized enrichment score (NES). The result represents how the activity of each CM is within a phenotype, therefore some CMs might be more active in males or females. In order to consider a CM different between sexes, we have established a threshold based on NES distribution (absolute NES > 2).

Biological pathways were discovered applying over-representation analysis (ORA) on identified CMs using the R package clusterProfiler (v4.0.5) (Yu et al., 2012). Gene Ontology (v7.5.1), KEGG (v7.5.1) and Reactome (v7.4) gene set collection for enrichment were obtained from MSigDB^2^, and the enrichment was considered significant if the Benjamini-Hochberg adjusted *p* < 0.0005 and the gene set included at least 10 genes. An interaction network was constructed by locating every gene in the PPI network obtained from the human dataset on StringDB (v11.5) (Szklarczyk et al., 2018). Genes with the highest number of interactions within a network are considered hubs. These genes were identified both in the co-expression network and in the PPI network by counting their number of interactions independently, and were labeled according to whether they were a hub in either network or in both.

### 2.3. Regulatory network inference

In order to build and analyze regulatory networks, three algorithms from the netZoo toolkit were used: PANDA, CONDOR and ALPACA. Gene regulatory networks were estimated using the Python (v3.7) implementation of PANDA (v0.8.1). PANDA gathers information from gene co-expression, PPI, and a prior TF motif-genes network to infer aggregate gene regulatory networks for each phenotype. Edge weights of PANDA networks are representative of the evidence of a regulatory interaction. PPI human dataset (v11.5) was inputted in the PANDA algorithm after genomic annotation conversion to Gene Symbol using clusterProfiler (Yu et al., 2012). The prior motif network was provided by PANDA developers^3^ and consisted of motif interactions of 730 TFs extracted from the Catalog of Inferred Sequence Binding^4^ (Weirauch et al., 2014). After removing the genes absent both in the motif dataset and in the gene expression dataset, the prior motif network contained regulatory information between 730 TFs and 15,150 genes. Only canonical TF-gene interactions were used in the analysis as recommended by Sonawane et al. (2017).

### 2.4. Regulatory network comparison

PANDA's male and female regulatory networks were initially submitted to the modularization algorithm CONDOR (v0.99.49) (Platig et al., 2016). CONDOR uses positive edge weights as evidence for the existence of an interaction between a TF and a gene, detecting bipartite community structures within each network using a maximum modularization approach (Barber, 2007).

Next, we used a graph-based approach called ALtered Partitions Across Community Architectures (ALPACA) (v0.99.49) (Padi and Quackenbush, 2018) that compares two networks and identifies *de novo* gene modules that best distinguish the networks. As described in the original article, ALPACA compares condition-specific networks to each other instead of to a random graph null model. In the comparison, one network is defined as the “baseline” network and the other is defined as the “perturbed.” ALPACA defines a score denominated the “differential modularity score” that compares the density of modules in the “perturbed” network to the expected density in the matched “baseline” network. In our case, it allows us to contrast networks of male and female samples and partition the nodes into optimal differential modules (Ms). Since the ‘baseline” community structure is the null model to which the “perturbed” network is compared, using the male network as baseline to compare to the female network could produce different results from the alternate comparison. For example, if the female network is used as the baseline, ALPACA compares the observed density of modules in the male network to the expected density in the female network, producing male-biased differential results. For this reason, we performed both analyses.

Differential modules were submitted to enrichment by ORA using the clusterProfiler package (Yu et al., 2012), and cut-off was set to Benjamini-Hochberg adjusted *p* < 0.05. Represented Gene Ontology (GO) terms were selected based on their uniqueness and dispensability determined by REVIGO, a tool that calculates redundancy between a list of GO terms and identifies the most representative ones (Supek et al., 2011). Based on the enrichment *p*-value of each GO term and its level of informativeness, REVIGO selects GO terms that best describes the clusters of similar semantic terms, generating a reduced GO term list. For our analysis, we chose to generate small lists as a result (REVIGO reduction parameter of 0.05). Next, we excluded from the analysis GO terms from tissues or biological processes not related to our samples, as well as those with other GO terms in the same ontological tree with lower significance scores. The whole GO module enrichments and REVIGO results are attached at the Supplementary materials, and excluded REVIGO terms are marked gray.

### 2.5. Extracting differential regulation drivers

ALPACA also estimates how much a TF or a gene contributes to the differences in module density, calculating its “differential modularity score.” Transcription factors with the highest score were extracted as described by Lopes-Ramos et al. (2021). Initially, scores were transformed to a log scale. The median and the interquartile range (IQR) for TFs and genes were calculated separately, given the structure of the bipartite network and number of interactions of each type of entity. TFs were considered significant if their differential modularity score deviated more than 1.5 × IQR from the module median they participated in, while genes were selected when their score deviated more than 3 × IQR.

### 2.6. Databases-specific enrichment analysis of ALPACA modules

Genes from each differential module (M) were submitted to a modular single-set enrichment test (MSET) (Eisinger et al., 2013) using the whole network as background. To determine the over-representation of genes from each differential module in specific cell types, enrichment analysis was performed with a mid-gestational developing fetal brain single-cell dataset, fit to the age of our samples (Polioudakis et al., 2019). Associated neurodevelopmental disorders and psychiatric disorders module enrichment was conducted using curated gene sets for Alzheimer's disease (AD) (Jansen et al., 2019), ADHD (Demontis et al., 2018), major depressive disorder (MDD) (Howard et al., 2019), bipolar disorder (Stahl et al., 2019), ID (Ilyas et al., 2020), syndromic and non-syndromic ASD genes from SFARI^5^, plus other independent ASD datasets (Sanders et al., 2015; Grove et al., 2019), schizophrenia (Lam et al., 2019), macrocephaly and microcephaly genes extracted from the testing panels by University of Chicago^6^ and Online Mendelian Inheritance in Man database^7^, and cross-disorders gene lists (Deciphering Developmental Disorders Study, 2017; Lee et al., 2019).

MSET enrichment was also performed to assess the role of gonadal hormones in the regulation of ALPACA modules. In order to obtain sex steroid hormone target genes, gene set collections for the TFs estrogen and androgen receptors (ER and AR, respectively) were obtained from MSigDB (v7.5.1, c3.tft.v7.5.1.symbols.gmt) (Liberzon et al., 2015). All 5 motifs for the main estrogen receptors (ER_Q6, ER_Q6_01, ER_Q6_02, TGACCTY_ERR1_Q2, ERR1_Q2) were joined, resulting in 1,534 ER target genes. Androgen receptor targets consisted of 537 genes, obtained by joining 5 AR motifs (AR_01, AR_02, AR_03, AR_Q2, and AR_Q6). The MSET algorithm was employed with 10,000 permutations and the *p*-value was generated based on the over-representation of the input gene list in the dataset of interest when compared to the random sets generated by the permutations. This empirical *p*-value was adjusted using the Benjamini-Hochberg method.

## 3. Results

### 3.1. Co-expression analysis reveals modules with sex-biased activity

Co-expression analysis with CEMiTool identified co-expression modules (CMs) of genes expressed from PCW 12 to 19 of the fetal brain development. These modules were also submitted to GSEA in order to determine sex activity and functional enrichment analysis based on Reactome, GO and KEGG.

Genes were grouped into 33 CMs based on the correlation of their normalized expression values, and 30 genes out of 18,904 were not correlated to any CM (Supplementary Figure S2). From all the CMs, a number of 13, 11 and 8 modules were significantly enriched for Reactome, GO and KEGG terms, respectively (adjusted *p* < 0.0005, and minimum of 10 genes) (Supplementary Table S2). Six out of 14 CMs that were considered different between sexes (absolute NES > 2) are significantly enriched for these databases (CM1, CM9, CM16, CM19, CM25, and CM31) (Supplementary Figure S3). Therefore, these following CMs will be discussed below.

Three selected modules were more active in females. CM1 is the largest module, composed of 5,306 genes. CM1 is more active in female samples (adjusted *p* < 0.001, NES = 2.35) and enriched for Reactome, GO and KEGG pathways associated with neurotransmitters, membrane receptors, ion channels, and signal transduction proteins, that act together in a complex molecular interaction network important to the synapses organization (Burke and Bender, 2019). This CM is also enriched for GO terms for cell maturation and neuron differentiation associated with synapses specification. CM16 (253 genes) is also more active in females (adjusted *p* < 0.001, NES = 2.88), and its genes are enriched for the citric acid cycle and respiratory electron transport pathways in both Reactome and GO databases. The last CM that is more active in females (adjusted *p* < 0.001, NES = 3.17), CM31 (78 genes) demonstrates significant enrichment for activation of HOX genes in hindbrain in the Reactome database, a term for which the module more active in males CM9 is also enriched.

**Figure 2:**
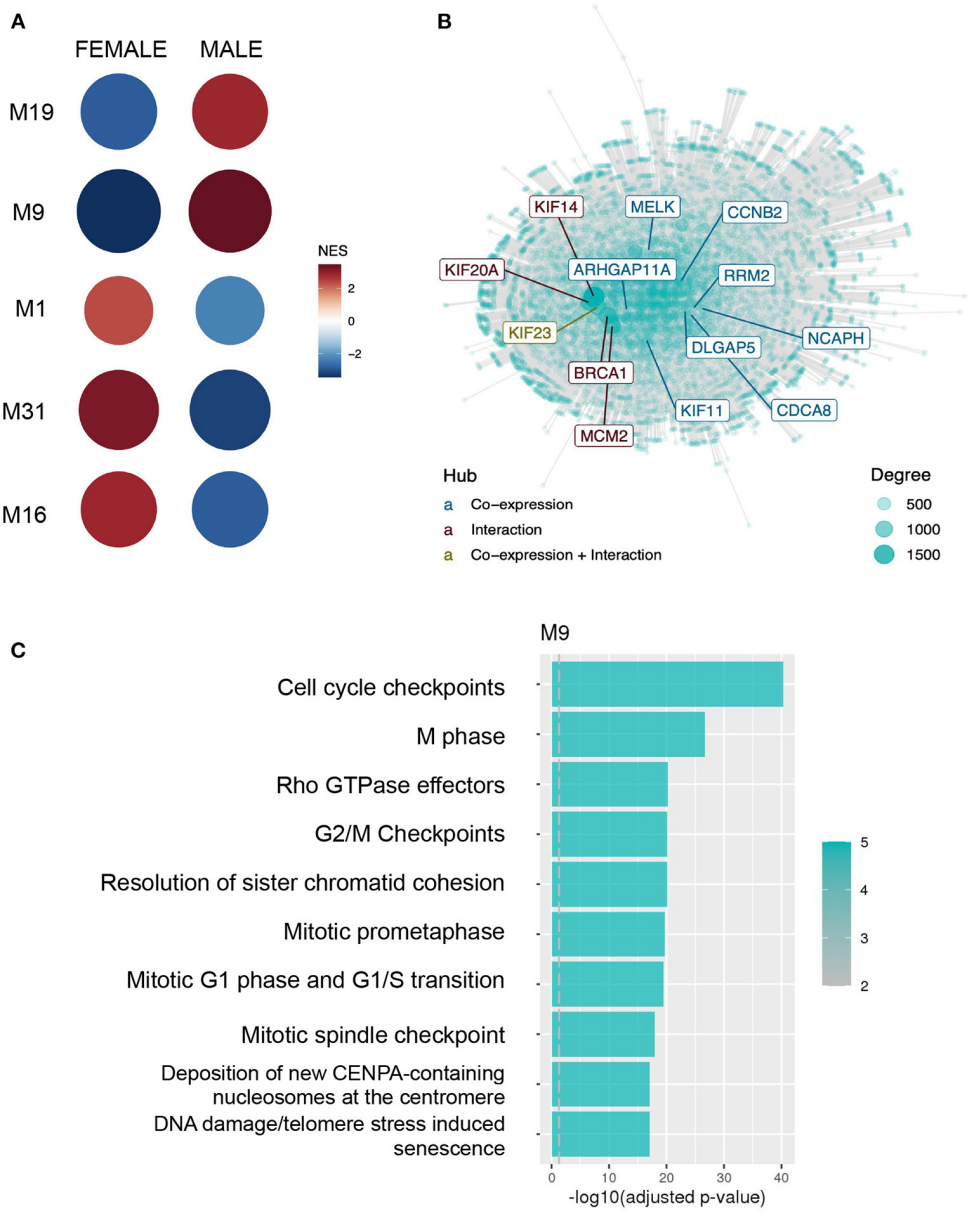
Normalized expression values from males and females were submitted to co-expression analysis with CEMiTool, demonstrating different patterns of expression associated with particular biological functions. **(A)** GSEA demonstrating each module activity for males and females. Circle color and size represents the Normalized Enrichment Score (NES). **(B)** Integrated network with interaction data for M9, with hub genes highlighted. **(C)** Over-representation analysis of genes from module M9 using pathways from the Reactome database.

The highest difference in sex activity (adjusted *p* < 0.001, NES = 3.43) was observed in CM9 (654 genes), more active in males. Hub genes tend to play important roles in the network. In CM9, genes like BRCA1, CCNB2, CDCA8, and genes from the KIF family are central (Figure 2A). Moreover, CM9 is significantly enriched for cell division processes, cell cycle repair and senescence and stress response according to all databases (see Figure 2B for Reactome results). Likewise, CM19 (229 genes) is more active in males (adjusted *p* < 0.001, NES = 2.83) and is consistently associated with ECM organization and ECM proteoglycans.

Altogether, CEMiTool results indicate 4 modules with sex differences in activity related to the following biological processes: (1) neuron differentiation and synapse specification; (2) mitochondria energy production; (3) cell proliferation and stress response; and (4) ECM organization. The first and second are more active in females, and the other 2 in males (Figure 2C).

### 3.2. Regulatory networks community structure comparison

We modeled male and female PANDA networks integrating gene expression, transcription motif and PPI data. These networks indicate how strong are the evidences of regulatory interactions between TFs and genes for each sex in the form of bipartite networks. Then, we identified community structures of male and female PANDA networks with CONDOR. In order to identify transitions in their regulatory network structures, ALPACA was run twice, alternating the sex network used as baseline. Therefore, differential networks using the female network as baseline will be referred to as female-baseline differential network, while results achieved with the male network as baseline will be referred to as male-baseline differential network. In our case, it is important to have in mind that the modularization of the female-baseline differential network result will give us, as explained on the Methods section, modules (Ms) that have a different density observed in the male network than the expected from the density in the female network. Therefore, it will give us male-biased modules and the associated results will be addressed as male-biased. Accordingly, using the male as baseline will give us differential network modules addressed as female-biased. Nevertheless, both results suggest differential patterns in gene regulation between male and female regulatory networks, but do not imply a direction in the difference like differential expression analysis or in activity like CEMiTool analysis. It also does not imply that a module is more or less regulated in any sex.

**Figure 3:**
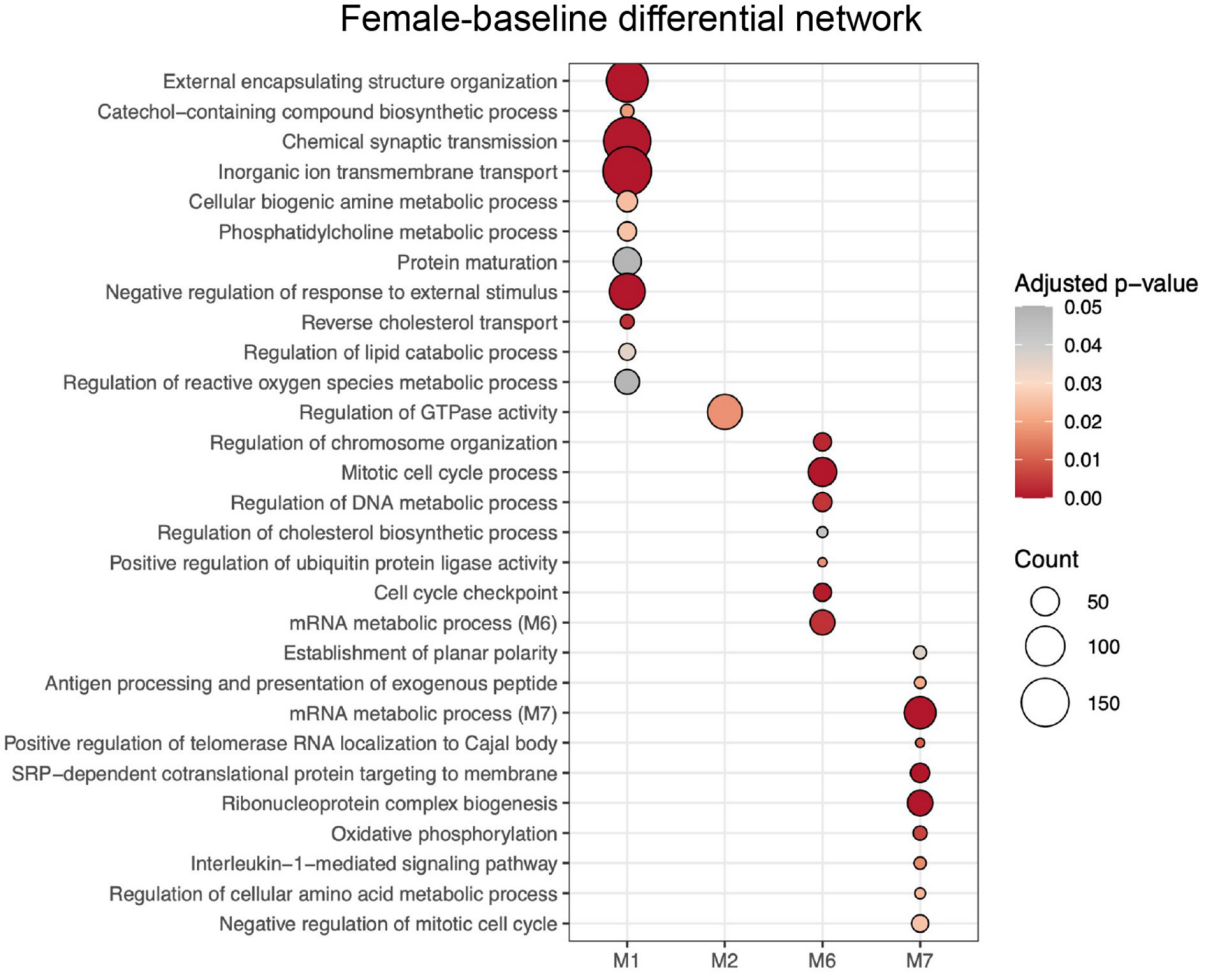
GO terms enrichment of modules from female-baseline differential network resulted from ALPACA analysis of differences in network topology from PANDA networks. GO terms were chosen according to their uniqueness, dispensability and significance based on REVIGO analysis. The color of the circle indicates the over-representation adjusted *p*-value, and the size represents the number of genes corresponding to the GO term.

ALPACA detected 7 differential modules when the female network was used as the baseline, which were determined male-biased modules (Supplementary Figure S4A). Each module was submitted to GO over-representation analysis using clusterProfiler, and 4 out of the 7 modules had significant enrichment (adjusted *p* < 0.05, gene set > 5) (Supplementary Table S3A). Next, we applied REVIGO to eliminate redundant GO terms, identifying a total of 118 GO terms that best described the 4 modules (Supplementary Table S3B). Male-biased M1 was enriched with GO terms associated with synapses (e.g. chemical synaptic transmission, inorganic ion transmembrane transport, and neurotransmitter transport); cell-cell adhesion, import into cell, cholesterol transport, regulation of lipid catabolic process, regulation of reactive oxygen species (ROS) metabolic process, extracellular matrix organization, cytokines, and inflammatory response; male-biased M2 with regulation of GTPase activity; male-biased M6 with different terms associated to cell cycle (e.g., mitotic cell cycle process, chromosome segregation, and cell cycle checkpoint signaling); and male-biased M7 with establishment of planar polarity, mitotic cell cycle, DNA repair, telomerase activity, mitochondrial translation, and gene expression. To visualize the results, we selected each male-biased module's top 10 REVIGO terms with the highest enrichment scores that best represented its category (Figure 3).

**Figure 4:**
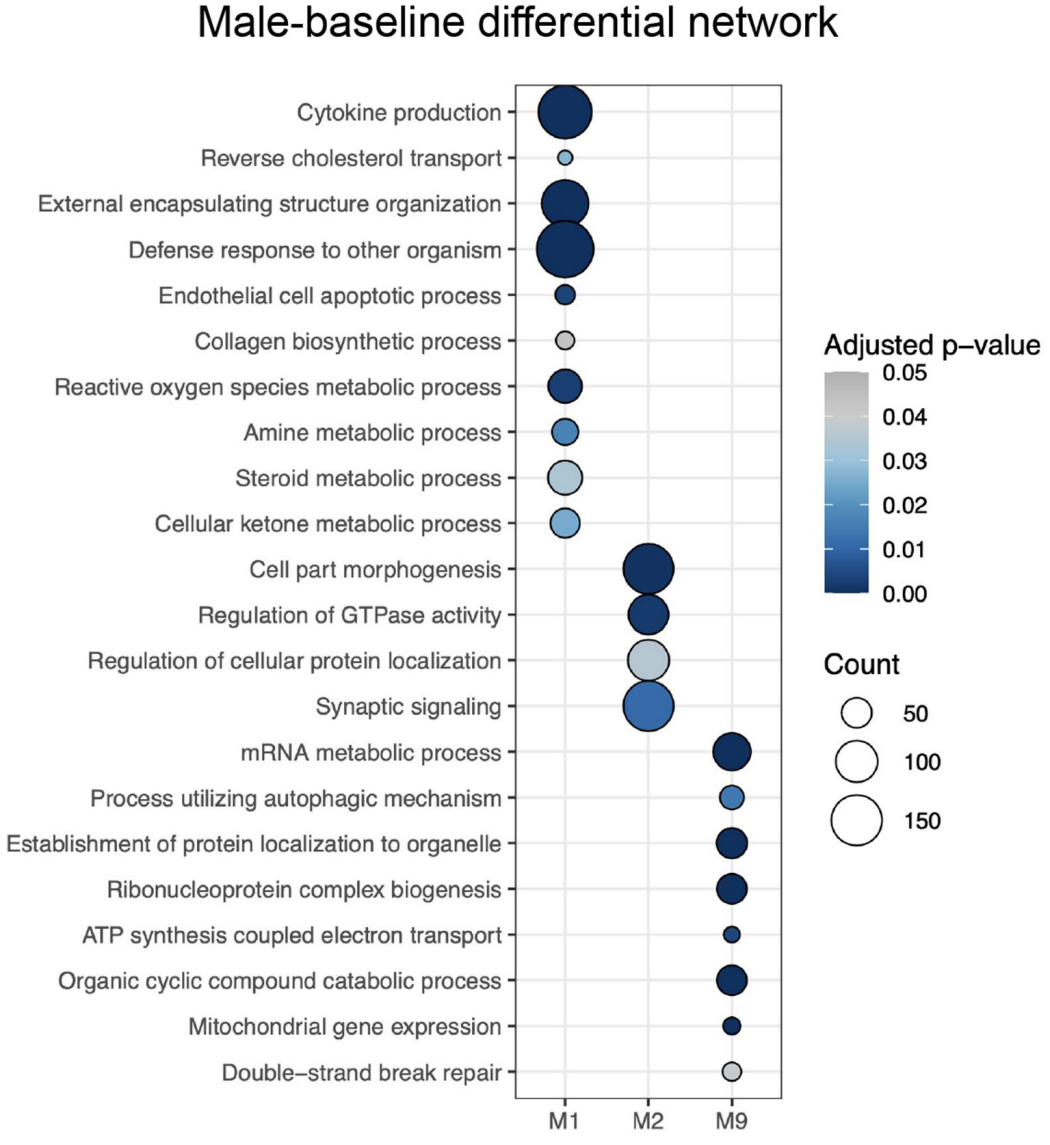
Enrichment of modules from the male-baseline differential network for GO terms selected by REVIGO. The intensity of the color of the circle indicates the over-representation adjusted *p*-value, and the size represents the number of genes corresponding to the GO term.

Meanwhile, male-baseline differential network ALPACA analysis revealed 10 differential modules, considered female-biased (Supplementary Figure S4B). Enrichment analysis revealed significant GO terms for 3 modules (adjusted *p* < 0.05, gene set > 5) (Supplementary Table S3C), and REVIGO reduced the number of terms to 84 (Supplementary Table S3D). Female-biased M1 was enriched with GO terms associated with cytokines production and immune system, reverse cholesterol transport, ROS metabolic process, metabolism (e.g., regulation of cellular ketone metabolic process, amine metabolic process, and regulation of nitric oxide metabolic process), extracellular modifications and tissue remodeling; female-biased M2 with GTPase activity and synaptic signaling; and female-biased M9 with process utilizing autophagic mechanism, and different terms associated with mitochondrial activity. The selected top 10 REVIGO terms can be seen in Figure 4.

In summary, ALPACA results point to regulatory differences between females and males in modules associated with: 1) synapse organization; 2) ECM modifications; 3) immune factors; 4) cell cycle and cell senescence; 5) metabolism and mitochondria function. It is important to note that the majority of these processes appear both in male- and female-biased modules, suggesting that they have differences in modularity comparing the observed density of nodes to the expected in both analyses. In addition, it is possible to observe that many of these biological processes were demonstrated to be enriched both in male- and female-biased modules, indicating that there are sex-specific differences in modularity involved in these differentially regulated processes.

### 3.3. Differential modularity core transcription factors and genes

The contribution of each TF and gene is calculated by ALPACA and defined as a score of “differential modularity.” Top TFs were defined as those which scores were 1.5 × IQR higher than the module median and top genes those which deviated 3 × IQR.

The female-baseline differential network comprises 24 top TFs and 3 top genes. These TFs and genes are associated with the modules that have a different density in the male network than the expected based on the female network, and thereby are classified as male-biased (Table 1). Most of the top TFs are from the Ets family, such as ELK1, ERF and ERG, all located in the male-biased M7. These TFs have been implicated in biological functions like cellular proliferation, differentiation, tissue remodeling, and apoptosis (Sementchenko and Watson, 2000). The top TF was ETV1, a protein that is associated with neuron cell differentiation (Flames and Hobert, 2009; Abe et al., 2012), connection formation (Arber et al., 2000), and is regulated by the androgen receptor (Cai et al., 2007). Some top TFs from the male-biased M6 are from the CREB and ATF families, which are involved in pathways of cAMP-dependent phosphorylative activation of multiple kinases that are pertinent to neuron proliferation, survival, maturation, differentiation, and synaptic establishment (Alberini, 2009; Landeira et al., 2016). Lastly, top TFs like NFYA, NFYB, NFYC, and GABPA, in addition to the top gene SARS2 are all relevant to mitochondrial function and lipid metabolism (Yokogawa et al., 2000; Yang et al., 2014; Benatti et al., 2015).

**Table 1:** Male-biased TFs and genes with the highest contribution to differential modularity.

**IDs**	**Score**	**Type**	**Membership**	**logScore**
ETV1	0.111032711911787	TF	7	–2.19793041917789
ETV3	0.105102133884857	TF	7	–2.25282269792897
ENSG00000235187	0.101989495776801	TF	7	–2.28288545358132
ELK1	0.100036227836841	TF	7	–2.3022228802326
ERF	0.0942194013630829	TF	7	–2.36212915933812
GABPA	0.0921935111193238	TF	7	–2.38386552921162
ERG	0.084327581715349	TF	7	–2.47304628225229
ELK4	0.0820083882832545	TF	7	–2.50093374081253
ELF4	0.0719844617675763	TF	7	–2.63130499203981
ETV2	0.0623491141159714	TF	7	–2.77500581520127
ETV6	0.0460089254404356	TF	7	–3.07891987000087
NFYB	0.0454872742240467	TF	6	–3.09032267952863
NFYC	0.0448559588330572	TF	6	–3.10429883791896
CREB3	0.0444155913215513	TF	6	–3.11416471529396
PBX3	0.0439291433374868	TF	6	–3.12517732181088
CEBPZ	0.0411715669742121	TF	6	–3.19000738285559
FOXI1	0.0411560241502577	TF	6	–3.19038496767076
ATF7	0.0407077302738889	TF	6	–3.2013372715492
NFYA	0.040400432360089	TF	6	–3.2089147920899
CREM	0.04007506050778	TF	6	–3.21700107062415
CREB1	0.0397179861278367	TF	6	–3.22595114280664
ATF1	0.039205356293987	TF	6	–3.23894190136741
CREB5	0.0384335142390451	TF	6	–3.25882543338601
ATF2	0.0375351526625051	TF	6	–3.28247738076136
SARS2	0.00667974832348833	Gene	6	–5.00867496826785
SCNM1	0.00610836004776335	Gene	7	–5.09809694644698
SF3A1	0.00580643130286095	Gene	7	–5.1487891304238

The male-baseline differential network contained 50 top TFs (Table 2) and 129 genes classified as female-biased (see Supplementary Table S4 for genes). Highest scored TFs are located at female-biased M9 and are essential for proper development of brain structures, mainly GSC (Parry et al., 2013), DMBX1 (Zhang et al., 2002), PITX3 (Verdin et al., 2014), and OTX1 (Larsen et al., 2010). Top TFs that belong to the female-biased M5 are from the bHLH superfamily that is related to neuronal differentiation regulation, such as proteins BHLHE23 and BHLHA15 (Skinner et al., 2010), oligodendrocyte transcription factors OLIG1, OLIG2, and OLIG3 (Jakovcevski, 2005), and neurogenic factors NEUROD2, NEUROG1, and NEUROG2 (Sun et al., 2001). Female-biased M3 contains TFs related to the Myc/Max/Mad network, extensively known for its role in regulating cell cycle, growth and survival (Grandori et al., 2000), such as MYC, MYCN, MNT, MLXIP, and MXI1. In addition, female-biased M3 top TFs NPAS2 and BHLHE40 are associated with the CLOCK gene pathway (Reick et al., 2001; Nakashima et al., 2008).

**Table 2:** Female-biased TFs with the highest scores of differential modularity.

**IDs**	**Score**	**Type**	**Membership**	**logScore**
GSC	0.15269444991972	TF	9	–1.87931641371986
DMBX1	0.143896164185085	TF	9	–1.93866332155886
PITX3	0.139609591555273	TF	9	–1.96890538359812
GSC2	0.136565488213182	TF	9	–1.99095101227904
PITX1	0.129696540209988	TF	9	–2.0425578633433
CRX	0.125931486465048	TF	9	–2.07201727813467
ATF7	0.123740411331103	TF	7	–2.0895693647285
CREB3	0.120790690784687	TF	7	–2.1136960594916
DPRX	0.120072842470422	TF	9	–2.11965669977571
CREB1	0.114068478575226	TF	7	–2.17095632167285
ATF2	0.111667400013813	TF	7	–2.19223046855887
CREB5	0.110148933472246	TF	7	–2.2059218882841
BHLHE23	0.108192582807058	TF	5	–2.22384246568603
BHLHA15	0.107423375532402	TF	5	–2.23097747131205
OLIG1	0.103916814738681	TF	5	–2.26416455817999
OLIG2	0.100935892899876	TF	5	–2.29326968742689
OLIG3	0.0992507778721316	TF	5	–2.31010552194296
CREM	0.0973928687453972	TF	7	–2.32900228718044
ATF1	0.0965080136608328	TF	7	–2.33812923096765
E4F1	0.0948727792385772	TF	7	–2.35521845077532
NEUROD2	0.0901541681065749	TF	5	–2.40623409516508
BHLHE22	0.0877130213855779	TF	5	–2.43368491416513
NEUROG1	0.0863604773712158	TF	5	–2.44922514566188
NEUROG2	0.0858153611956347	TF	5	–2.4555572535989
BATF3	0.0752807014700203	TF	7	–2.58653146560257
ETV1	0.0736046279099473	TF	8	–2.60904737601288
ETV3	0.0732706608698659	TF	8	–2.61359501119943
ENSG00000235187	0.0728467393263581	TF	8	–2.61939750607484
ELK1	0.0726022269170718	TF	8	–2.62275968383295
TCF23	0.0677728510262973	TF	5	–2.69159359156897
ERF	0.0659989002319932	TF	8	–2.71811720024616
GABPA	0.0653488923062923	TF	8	–2.72801478922706
TWIST2	0.06246467706455	TF	5	–2.77315404897403
ERG	0.0577576316972216	TF	8	–2.85149978771012
ELK4	0.0572357326313101	TF	8	–2.86057687928635
MNT	0.0557028778394643	TF	3	–2.88772346660684
JUN	0.0555297227080979	TF	7	–2.89083685729236
MLXIP	0.0541104351118061	TF	3	–2.9167282261141
ELF4	0.0533629664474969	TF	8	–2.93063828582599
ID2	0.0516803037243714	TF	3	–2.96267854250549
OTX1	0.0515334369812805	TF	9	–2.96552442021925
NPAS2	0.0510333269816641	TF	3	–2.9752763894478
MXI1	0.0479890121591645	TF	3	–3.03678320762965
MYCL1	0.0457721650316248	TF	3	–3.08407912301834
MYCN	0.0446185170191288	TF	3	–3.10960632631831
ETV2	0.0398554499721332	TF	8	–3.22249612093557
ELF1	0.0391257977816137	TF	8	–3.24097323974533
MYC	0.0381768714055192	TF	3	–3.26552540732339
HEY1	0.0381381103891828	TF	3	–3.26654122410688
BHLHE40	0.037927235388315	TF	3	–3.27208581314271

Interestingly, 13 TFs have a high score of differential modularity in both female- and male-baseline differential networks (Supplementary Table S5). Aforementioned top TFs from the Ets family are important to the female-biased M8 in a similar fashion to male-biased M7. Likewise, TFs from the CREB and ATF families are clustered in the male-biased M7, repeating their pattern in the female-biased M6. Integrating these results with CEMiTool co-expression modules, we observe that some of the shared top TFs are allocated in modules with sex differences in activity (Supplementary Table S5). The top TF ERF belongs to the CM9, which is more active in males and related to cell cycle control, in consonance with the female-biased M7 functional enrichment. In addition, top TFs ATF1, ATF2, and ETV1 are co-expressed in CM2, more active in males, while ATF7 and CREB5 are co-expressed in CM1 and more active in females.

### 3.4. ALPACA's differential modules enrichment for databases of interest

**Figure 5:**
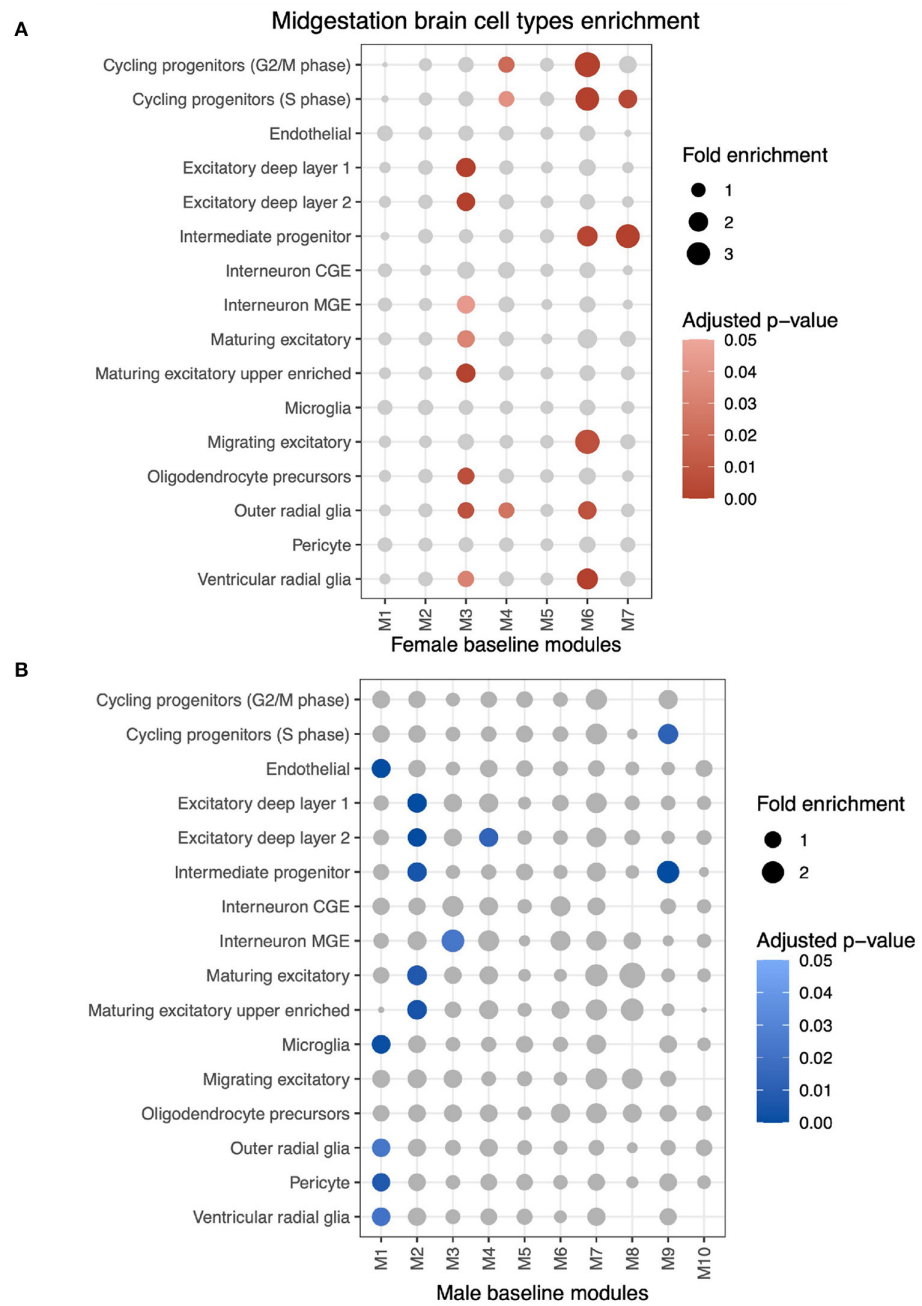
MSET analysis of all genes from differential modules for enrichment of mid-gestational cell types identified by fetal brain single-cell RNA-seq. **(A)** MSET analysis of genes from differential modules using the female network as the baseline. Circle size indicates module gene fold enrichment of the cell type dataset and significant enrichment (adjusted *p* < 0.05) is represented by the red scaled colors. **(B)** Cell type results using the male differential network.

Using MSET, each ALPACA's differential module was tested for enrichment to genes expressed in mid-gestational cell types (Figure 5 and Supplementary Table S6A). Since our dataset consists of non-dissected bulk RNA-seq, these results indicate that biological processes that are characteristic of certain cell types could be differentially regulated in ALPACA's differential modules.

Genes from female-biased M1 are enriched for endothelial (fold enrichment (FE) = 1.38; adjusted *p* < 0.001), microglial (FE = 1.35; adjusted *p* = 0.001), pericyte (FE = 1.24; adjusted *p* = 0.007), outer radial glial (FE = 1.24; adjusted *p* = 0.002), and ventricular radial glial (FE = 1.31; adjusted *p* = 0.002) cells. This ALPACA module was associated with cytokine production, endothelial cell apoptotic process and tissue remodeling biological processes.

Female-biased M2 and male-biased M3 are both enriched for genes expressed in differentiated or maturing excitatory neurons, which are generated during neurogenesis (Taverna et al., 2014). Female-biased M2 is also enriched for GO terms such as synaptic signaling and cell part morphogenesis, which are representative of neuronal activity in differentiated neurons and of dendritic and axonal growth in maturing neurons.

Another shared cell type enrichment pattern occurs in male-biased M7 and female-biased M9 for cycling progenitors (S phase) (FE = 1.8; adjusted *p* = 0.003, and FE = 1.58; adjusted *p* = 0.012, respectively) and intermediate progenitor (FE = 2.08; adjusted *p* < 0.001, and FE = 3.14; adjusted *p* < 0.001, respectively). These modules are associated with energy production and cell cycle regulation.

The cell cycle related male-biased M6 is enriched for genes expressed in outer radial glia (FE = 1.72; adjusted *p* = 0.008) and ventricular radial glia (FE = 2.37; adjusted *p* < 0.001), which are proliferative cells, as well as with cycling progenitors from G2/M (FE = 3.51; adjusted *p* < 0.001) and S phase (FE = 3.06; adjusted *p* < 0.001) and intermediate progenitors (FE = 2.22; adjusted *p* = 0.003). This enrichment pattern does not repeat in any female-biased module. Overall, cell type gene expression enrichment correlates with biological processes enriched within the same ALPACA module.

**Figure 6:**
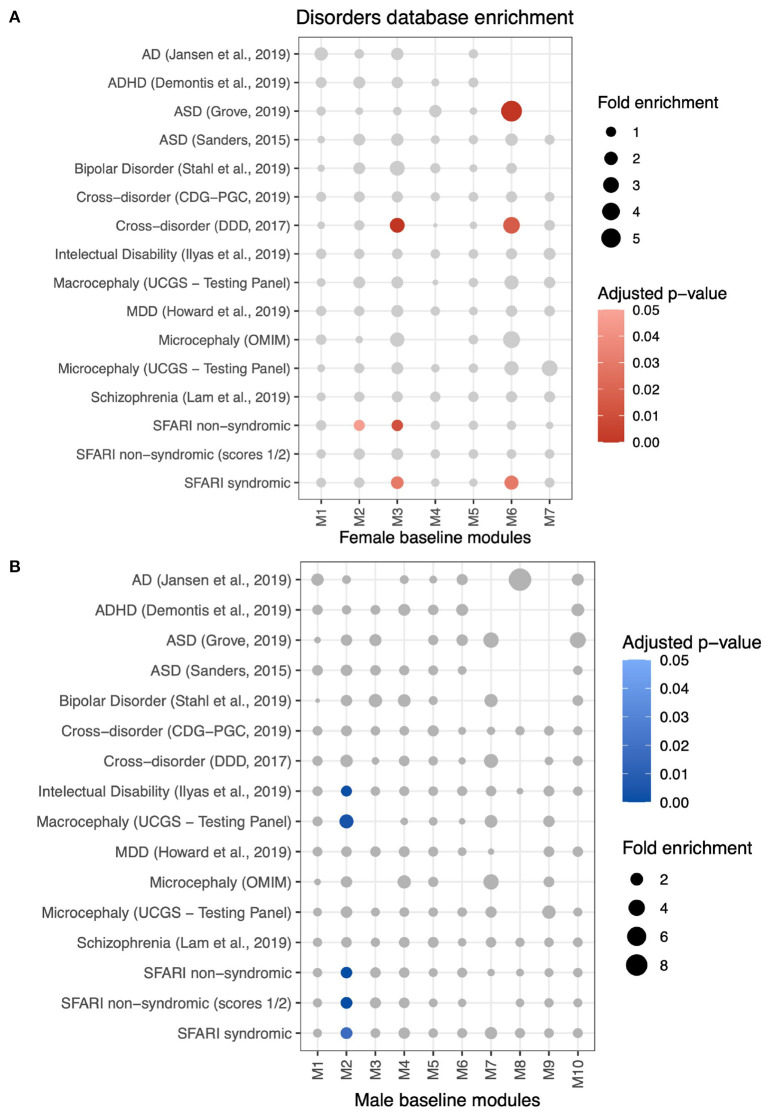
Enrichment analysis using MSET to identify differential modules with genes associated with neuropsychiatric disorders lists from multiple databases (see Methods). **(A)** Disorders to which female-baseline differential modules are significantly enriched are represented by red scaled circles (adjusted *p* < 0.05). Circle size is relative to the fold enrichment. **(B)** Enrichment of male-baseline differential modules to disorders databases.

We also aimed to discover whether genes grouped in differential modules with significant enrichment for biological processes pertained to gene lists associated with neuropsychiatric disorders (Figure 6 and Supplementary Table S6B). MSET analysis results show that female-biased M2 is enriched for intellectual disability (FE = 1.31; adjusted *p* = 0.001), macrocephaly (FE = 2.63; adjusted *p* = 0.004), non-syndromic ASD genes from SFARI (FE = 1.5; adjusted *p* < 0.001), non-syndromic ASD genes with scores 1 or 2 (high confiability) (FE = 1.61; adjusted *p* < 0.001), and syndromic ASD genes (FE = 1.64; adjusted *p* = 0.017). Moreover, male-biased M6 is enriched for ASD genes (FE = 5.94; adjusted *p* < 0.001), genes in the cross-disorder database (FE = 3.47; adjusted *p* = 0.016), and syndromic ASD genes (FE = 2.26; adjusted *p* = 0.03).

Lastly, we analyzed our ALPACA differential modules for enrichment of gene targets of sex steroid hormone receptors (Supplementary Table S6C). Male-biased modules M1 and M6 were enriched for estrogen receptor (ER) targets (FE = 1.27; adjusted *p* < 0.001, and FE = 1.46; adjusted *p* = 0.01, respectively). For female-biased modules, only M2 was enriched for ER targets (FE = 1.29; adjusted *p* < 0.001). Curiously, none of the modules were enriched for androgen receptors.

## 4. Discussion

Our study used integrative approaches that account for biological complexity and takes advantage of its interacting entities to better understand sex differences in brain autosomal gene expression regulation. In this context, we applied network tools that take advantage of community information, since methods that identify changes in groups of nodes rather than individual edges or nodes have been demonstrated to find more robust differences between distinct phenotypes (Padi and Quackenbush, 2018). CEMiTool clusters genes based on the correlation of their expression, and could identify co-expression modules differentially activated in two conditions. Co-expressed genes often have related biological functions or participate in the same regulatory pathways (Langfelder and Horvath, 2008). Co-expression networks are built as a consequence of gene expression regulation, however they do not convey direct information about regulatory interactions. Therefore, we combined co-expression networks with regulatory networks inferred by the netZoo tools in order to extract this information. These complementary analyses also increase the reliability of our results that are achieved by the two independent tools, and thereby we expect similar biological processes to be pointed out as differentially co-expressed and regulated between sexes in both analyzes. Moreover, we observe that these differences in the regulation of biological processes are a consequence of the sex differences in modularity, and the addition of the CEMiTool results shows in which sex this biological process is more active.

In a recent review, Kostović et al. (2019) divided the fetal and perinatal developmental stages characterized by the occurrence and intensity of major histo- and neurogenetic events, originating transient patterns of microstructural features that will later differentiate into the multiple areas of the brain. The early fetal period (8–12.5 PCW) is characterized by three histo- and neurogenetic events: proliferation, migration and molecular specification, culminating in the establishment of the cortical plate. From 13 to 15 PCW, these events continue while cells begin to change aggregation patterns and migrate tangentially, causing the dispersion of cortical plate cells, reducing its density, and ultimately forming the cortical subplate. Through the midfetal period (15–23 PCW) four events are dominant: neuronal aggregation and cytoarchitecture changes, axonal outgrowth and ingrowth, dendritic differentiation, and molecular specification. Neuronal proliferation continues in the ventricular zones, and new postmitotic neurons migrate through the intermediate zone and the subplate, defining transitory patterns of lamination and of microstructural organization of subcortical areas. Moreover, neuronal differentiation, dendritic development, astroglia differentiation, gliogenesis and synaptic activity are also observed during the midfetal period.

In this context, this study contributes to the understanding of sex bias in brain development by capturing differences in pathways and biological processes in accordance with the brain development events that have been described for the fetal period of our samples, which range from 12 to 19 PCW of age. Collectively, CEMiTools, and netZoo results showed sex differences mainly in processes associated with progenitor proliferation and survival as well as cell fate decisions pertinent to cell delamination and differentiation, cell migration and sinaptogenesis.

Sex differences in cell proliferation is a consistent result along the conducted analyses. ALPACA's male-biased M6 is associated with cell cycle regulation while no female-biased module is, indicating that the regulatory patterns of this function in females are organized differently in males. This same module is enriched for multiple proliferative cell types as demonstrated by MSET analysis using mid-gestational brain gene sets, corroborating that these regulatory interactions are relevant to the neural progenitor activity and contribute to sex differences. Complementarily, CEMiTool demonstrates that cell cycle associated genes present the highest difference in activity between sexes, with higher mean expression in males. It has been demonstrated that prenatal brain growth in males is greater than in females from very early periods, and fetal head circumference measures are consistent with this concept (Gutiérrez-Adán et al., 2006; Broere-Brown et al., 2016). This higher proliferative profile has been well observed in cancer, resulting in higher incidence and mortality in males (Rubin et al., 2020). Abnormal neuronal proliferation has also been associated with NDs more common in males, such as ASD and ID (Ernst, 2016; Marchetto et al., 2016). Accordingly, male-biased M6 genes are enriched for two ASD gene sets, suggesting that this differential regulation in cell proliferation could be related to the male-bias in ASD.

We have also observed cohesive sex differences in the regulation of metabolism. Our discoveries show that genes related to the citric acid cycle, oxidative phosphorylation, and ATP synthesis are differentially regulated, while also more active in females. Metabolic processes are intrinsically involved in brain development because they regulate energy homeostasis, mediating nutrient availability, stress response and hormonal stimuli, and cell growth and division (Fritz and Fajas, 2010). The proliferative state of cells during neurogenesis is correlated with a decreased oxidative phosphorylation metabolism and an increase in the glycolytic activity, which is less efficient but produces ATP faster allowing for the augmented biosynthesis of macromolecules (Ježek et al., 2010). The glycolytic pathway includes the oxidation of fatty acids, which is a signal to induce cell stemness (Knobloch, 2017). It has been demonstrated that in normal conditions males favor the glycolytic pathway to generate ATP quickly, increasing their glucose and pyruvate uptake and producing more lactate, while females favor the pentose phosphate pathway (Rubin et al., 2020). Previous findings demonstrated that given an abundance of nutrients, normal proliferating cells can use glucose and glutamine as substrates to produce ATP and maximize their growth *via* PI3K/mTOR signaling pathways (Ray et al., 1995; Heiden et al., 2009). Research with murine hearts demonstrate that the PI3K/mTOR pathways are sexually dimorphic (Gürgen et al., 2013). TP53 gene regulation is more active in males according to our results. It is intricately associated with our top TFs in protein-protein interaction networks, and together with the top TFs from the MYC family it plays an important role in the glycolytic pathway (Whan Kim et al., 2004). MYC genes are known to stimulate glucose uptake, NADPH biosynthesis for redox homeostasis and glycolysis (Dang, 2010). In fact, the motif for MYC-associated zinc finger protein has been shown to be extremely differentially targeted between sexes across multiple tissues even when there are no differences in targeted gene expression (Lopes-Ramos et al., 2020). Therefore, we provide additional evidence to the theory that males naturally present a more proliferative metabolic profile, which seems to be partially defined by the activity of TFs from the MYC family.

Our results also point to a sex biased regulation of lipid catabolic processes, reverse cholesterol transport and amine and steroid metabolism. These lipid metabolic processes are essential for neural progenitor survival, proliferation and differentiation, in addition to ROS homeostasis, and mitochondrial biogenesis and function (Fame and Lehtinen, 2021). Cholesterol, for instance, is one of the most abundant lipids in neuron membranes and in the myelin sheaths, being essential for proper synaptogenesis, brain development and all sex steroid hormones biosynthesis (Orth and Bellosta, 2012). Consequently, the loss of the capacity to produce cholesterol in murine neural stem cells induces apoptosis, halting proliferation (Saito et al., 2009). Neural stem cells metabolism is primarily glycolytic, shifting to oxidative as they mature, and higher lipogenesis activity is essential to neuronal differentiation since lipids are necessary for the constitution of the membrane of neurons, their projections and vesicles (Knobloch, 2017). Lipogenesis and nucleotide synthesis are increased with the redirection of the glucose carbon flux to anabolic pathways caused by aerobic glycolysis, a process regulated by one of the top TFs we encountered, MLXIP (Fritz and Fajas, 2010). Indeed, females prioritize lipidic pathways, synthesizing and consuming more lipids (Ockner et al., 1979). These sex differences in cell metabolic profiles could be related to the higher proliferation of neural stem cells in males, while females shift earlier to an oxidative profile that differentiates progenitor cells into mature neurons. It is also notable that sex steroid hormones are intricatedly involved in metabolic differences through cAMP signaling pathways (Mayes and Watson, 2004), and their role in neuronal proliferation and differentiation has been extensively described (Karaismailoglu and Erdem, 2013; Miranda and Sousa, 2018).

In addition to the aforementioned metabolic functions of mitochondria in energy metabolism, apoptosis regulation and steroid hormone production, ALPACA also indicates regulatory differences in terms related to mitochondrial overall activity (mitochondrial gene expression, mRNA metabolic process, and mitochondrial translation) represented mainly in female-biased ALPACA module M9. Indeed, females have higher mitochondrial activity than males in blood and muscle cells (Cardinale et al., 2018; Silaidos et al., 2018), and CEMiTool reveals that related genes are more active in female fetal brain samples as well. Studies demonstrated that mitochondria from females produce more antioxidant proteins, therefore accumulating less ROS (Borrás et al., 2003). In consonance, glucocorticoid mediated ROS increase during gestational stress is higher in males, and sex-specific placental adaptation makes male fetuses keep growing even in adverse situations (Stark et al., 2011). ROS can have multiple sources in cells, including the activity of endothelial nitric oxide synthase, xanthine oxidase, membrane bound NADPH-oxidase and from the electron transport chain (Nayernia et al., 2014), which appeared in our results as differentially regulated. Mitochondrial functions have been associated with the coordination of cell proliferation, differentiation, migration and maturation in corticogenesis (Fame and Lehtinen, 2021), and ROS dynamics are essential to regulate cell fate (Inoue et al., 2016). Indeed, altered metabolic activities of ROS during neurogenesis are associated with neurodevelopmental disorders such as schizophrenia (Paulsen et al., 2012).

There is evidence that aerobic glycolysis reduces ROS levels, and that their interplay is related to cell fate decision with the participation of MYC proteins (Rodic and Vincent, 2017). These intricate interactions are signaling factors for proliferation, differentiation, apoptosis, and autophagy when ROS is overabundant (Reczek and Chandel, 2017). Autophagic mechanisms appear as differentially regulated, and associated with female-biased modules. Male cells are normaly more autophagic than female cells during the prenatal period (Addis et al., 2014), and autophagy has been suggested to be regulated by sex dimorphic pathways such as PI3K/mTOR and MAPK (Shanware et al., 2013). At least in parts, these differences are attributed to differential regulation of the insulin-growth factor 1 (IGF-1), which has been demonstrated to be regulated in sexually dimorphic fashion and increases glucose uptake (Clifton et al., 2010). Our results suggest that this balance is differentially regulated between sexes, as seen in the enriched functions of female-biased M7 and male-biased M9. These modules are also enriched for several types of progenitor cell corroborating that their genes are relevant to proliferative functions. Altogether, these results could indicate that this complex metabolic conjecture is underlying higher male susceptibility to NDs in complicated gestations.

ALPACA's female-biased module M2 is associated with cell part morphogenesis and synaptic signaling, which are functions related to tissue remodeling. During the morphogenesis of the cerebral cortex, multiple cytoarchitecture transient structures are shaped and subsequently rearranged due to neural progenitor cells proliferation, migration and differentiation, culminating in tissue histogenesis (Kostović et al., 2019). As reviewed by Sokpor et al. (2022), this process requires delamination and depends on the dismantling of adherens junctions, modification of cell polarity, cytoskeleton remodeling, and reposition of organelles and signaling molecules after mitosis. Therefore, changes in ECM dynamics are indispensable to the regulation of neurogenesis, and improper control of these events affect brain development (Long and Huttner, 2022). In fact, delamination is suggested to be the most important mechanism to allow for cell migration and differentiation in the corticogenesis (Sokpor et al., 2022). ECM components are also relevant to neurogenesis due to their pro-proliferative signals through cell-cell integrins interaction (Kalebic and Huttner, 2020). Interestingly, CEMiTool module CM19 is more active in males and associated with ECM. Male-biased gene expression in developing human brain samples has been linked to ECM genes, which have been implicated in the etiology of autism (Ziats and Rennert, 2013). Both CEMiTool and ALPACA results corroborates sex differences in functions pertinent to these ECM related processes, such as ECM organization, regulation of cell-cell adhesion, establishment of planar polarity and tissue remodeling. Sex differences encountered in synaptic signaling and immune response might reflect the relevance of neurotransmitters and cytokines as signaling molecules that control processes during brain development (Deverman and Patterson, 2009; Ojeda and Ávila, 2019). These processes are enriched in both female- and male-biased ALPACA modules and are more active in females in the CEMiTool module CM1. Neurotransmitters are essential for cell migration due to its chemotropic effects on migrating neurons (Behar et al., 1996), and there is abundant evidence that neurotransmitters and their receptors modulate gene expression through calcium and CREB signaling pathways impacting on neurogenesis, neuronal differentiation and programmed cell death (Jagasia et al., 2009; Ascenzi and Bony, 2017). Studies with animal models have demonstrated neurochemical differences between males and females, mostly due to sex hormones action (Uhl et al., 2022), and our results corroborate the hypothesis that during corticogenesis estrogen is a relevant signaling molecule (Denley et al., 2018). In a similar fashion, cytokines are critical signaling factors for the renewal of neural stem cells, regulation of differentiation between neurons and glial cells, determination of neuronal identity, and chemotropism, modifying cell migration and axon guidance (Deverman and Patterson, 2009). One of the differentially regulated cytokines between males and females we identified, interleukin-6 is temporally and spatially controlled in order to guarantee proper brain development, participating in multiple pathways of neuronal survival and differentiation (Kummer et al., 2021). For instance, this cytokine can promote ambiguous results: activating STAT-3 pathway, it induces gliogenesis, and through the MAPK/CREB pathway it can stimulate neurogenesis (Erta et al., 2012). Interestingly, women carrying female fetuses produce more IL-6, TNF-α and IL-1β (Mitchell et al., 2017).

A previous study with the same dataset identified differences in sex expression (O'Brien et al., 2018), and demonstrated that genes more expressed in males are enriched in neural progenitor cells marker genes, while genes more expressed in females are enriched in Cajal-Retzius cells and glia. We have identified enrichment for neural progenitors and glial cells in sex-biased modules, but Cajal-Retzius cells markers were not included in the single-cell database. However, we would like to highlight that unlike in differential expression analysis, sex-biased modules only indicate that regulatory patterns are different and do not convey a direction of the differences. For this reason, we can not assume that any sex is enriched for any cell type, but we can infer that sex differences in regulatory patterns are relevant to cell type processes. Moreover, the differences between the two studies could also be attributed to methodological differences and the usage of distinct databases.

The same limitation applies to the enrichments found for genes in disorders databases. A transcriptomic study identified that genes that are more expressed in fetal male brains are enriched for disorders like schizophrenia, Alzheimer's disease and autism (Shi et al., 2016). In our analyses, we found sex differences in regulatory patterns of modules enriched for intellectual disability and mainly ASD. Once again, our methods do not imply that the enrichment is specific to any sex. Besides, our dataset contains a larger number of samples and a narrower and more specific time window of the fetal period, which could explain why some enrichments were not reproduced.

Altogether, our study provides evidence that autosomal genes related to biological functions pertinent to neurodevelopment are differentially regulated between sexes. The mechanisms that are possibly responsible for these differences are the gonadal hormones, the sex chromosomes, and the sex-specific epigenetic events (McCarthy, 2016; Maschietto et al., 2017; Tahira et al., 2018). Our findings reinforce that CREB and MYC participate directly in the pathways that differentiate males from females during development (Auger, 2003), and that gene expression regulation of genes involved in metabolism, proliferation, and delamination contribute to neurodevelopmental differences between males and females. Those processes are associated in a complex and intricate mechanism relevant to neurodevelopmental disorders sex bias, suggesting that multiple factors with evidences of sex differentiation interact and regulate development. Important limitations from this study are the usage of bulk tissue samples and a temporal interval, which narrow the spatial and temporal understanding of the development dynamics, and the fact that our results are inferences based on bioinformatics tools and molecular biology assumptions, requiring further exploration of these regulatory interactions with practical experiments. Nevertheless, we believe our study lays a path for the connection of sex differences in multiple pathways with interactions that had not been established yet. These findings could help to understand how these differences in brain development impact on sex-biased neuropsychiatric disorders and provide targets for sex-specific terapeutic interventions.

## Data availability statement

Publicly available datasets were analyzed in this study. This data can be found here: https://doi.org/10.6084/m9.figshare.6881825.v1. No new custom software or code has been used in this paper. Codes (R and Python programming language) are all based on pre-existing, peer-reviewed published softwares for RNA-Seq and network analyses, as described above. These codes are available at https://github.com/victortoledo/frontiers_sexdiff_fetal_brain.git, along with some of the multiple data that were integrated, in order to ensure clarity and reproducibility.

## Author contributions

VT, AT, and HB designed the study and interpreted the results. VT, AF, AB, and AT conducted the analyses. VT and HB drafted the manuscript. All authors contributed with the further writing of the manuscript and approved the final manuscript.

## Funding

This study was financed by Fundação de Amparo à Pesquisa do Estado de São Paulo (FAPESP 2018/18560-6) and Coordenação de Aperfeiçoamento de Pessoal de Nível Superior—Brasil (CAPES)—Finance Code 001.

## Conflict of interest

The authors declare that the research was conducted in the absence of any commercial or financial relationships that could be construed as a potential conflict of interest.

## Publisher's note

All claims expressed in this article are solely those of the authors and do not necessarily represent those of their affiliated organizations, or those of the publisher, the editors and the reviewers. Any product that may be evaluated in this article, or claim that may be made by its manufacturer, is not guaranteed or endorsed by the publisher.

## Footnotes

^1^ https://www.genenames.org

^2^ http://www.gsea-msigdb.org/gsea/msigdb/collections.jsp

^3^ https://sites.google.com/a/channing.harvard.edu/kimberlyglass/tools/resources

^4^ http://cisbp.ccbr.utoronto.ca

^5^ https://gene.sfari.org

^6^ https://dnatesting.uchicago.edu

^7^ https://omim.org

## Supplementary material

Supplementary Figure S1 Singular Value Decomposition Analysis (SVD) of top principal components correlated to the samples covariates before and after batch correction using LIMMA, demonstrating the relevance of the batch effect for data variance.

Supplementary Figure S2 CEMiTool co-expression modules and their sizes. Not correlated genes are represented by the bar at the bottom.

Supplementary Figure S3 Sex activity of genes from each of the modules of co-expression detected by CEMiTool. Modules with a significant enrichment (adjusted *p* < 0.05) of gene expression of either sexes calculated by GSEA and an absolute NES > 2 are highlighted and are considered relevant. In red, modules that are more active in females; in blue, modules more active in males.

Supplementary Figure S4 ALPACA's female- and male-baseline differential module sizes and its composition by TFs (cyan) and genes (purple).

Supplementary Table S1 Covariates and principal components associated with samples as described by O'Brien and Bray (2018).

Supplementary Table S2 **(A)** CEMiTool module enrichment by over-representation analysis using Reactome pathways as background. **(B)** CEMiTool module enrichment by over-representation analysis using GO terms as background. **(C)** CEMiTool module enrichment by over-representation analysis using KEGG pathways as background.

Supplementary Table S3 **(A)** ALPACA's male-biased modules enrichment by over-representation analysis of GO terms. **(B)** GO terms selected with REVIGO using genes from ALPACA's male-biased modules. **(C)** ALPACA's female-biased modules enrichment by over-representation analysis of GO terms. **(D)** GO terms selected with REVIGO using genes from ALPACA's female-biased modules.

Supplementary Table S4 Female-biased TFs and genes with the highest contribution to differential modularity.

Supplementary Table S5 Shared top TFs between male- and female-biased regulation modules and their membership in CEMiTool co-expression modules.

Supplementary Table S6 **(A)** MSET enrichment results for ALPACA's differential modules using mid-gestational cell type gene lists as background. **(B)** MSET enrichment results for each ALPACA's differential modules using neuropsychiatric disorders associated genes as background. **(C)** MSET enrichment results for ALPACA's differential modules using lists of gene targets of sex steroid hormones receptors as background.

## Abbreviations

NDs: neurodevelopmental disorders
ID: intellectual disability
ASD: autism spectrum disorder
ADHD: attention deficit hyperactivity disorder
TFs: transcription factors
PPI: protein-protein interaction
M: module
PCW: post-conception weeks
VST: variance-stabilizing transformation
GSEA: gene set enrichment analysis
NES: normalized enrichment score
ORA: over-representation analysis
GO: Gene Ontology
IQR: interquartile range
MSET: modular single-set enrichment test
AD: Alzheimer's disease
MDD: major depressive disorder
ER: estrogen receptor
AR: androgen receptor
ECM: extracellular matrix
ROS: reactive oxygen species.
